# Indirect comparison between powered and manual circular staplers for left-sided colorectal anastomoses: clinical and economic outcomes in China

**DOI:** 10.1186/s12962-022-00380-1

**Published:** 2022-08-31

**Authors:** Junwei Bai, Yingnan Zhao, Hong Liang, Junmeng Li, Chao Zhang

**Affiliations:** 1 Department of Gastrointestinal Surgery, Henan Provincial People’s Hospital, Zhengzhou, Henan People’s Republic of China; 2 Division of Clinical and Administrative Sciences, College of Pharmacy, Xavier University of Louisiana, New Orleans, LA USA

**Keywords:** Clinical outcomes, Budget impact analysis, Powered circular stapler, Colorectal anastomoses, Real-world evidence

## Abstract

**Aims:**

This study aimed to examine the economic and clinical benefits of a new powered circular stapler for left-sided colorectal construction in China.

**Methods:**

A decision analysis model was constructed for a cohort of adult patients who underwent left-sided colorectal anastomoses, using either the Echelon Circular Powered (ECP) stapler) or the conventional manual circular staplers (MCS). The complications rates and healthcare resource utilization in the ECP cohort were obtained from the single-arm ECP trial (NCT03326895). For the MCS cohort, retrospective data from 20 Chinese hospitals were analyzed. Listing prices were used to estimate the costs of the staplers in China. Propensity score matching (PSM) was employed to adjust for the imbalance between the two cohorts. Anastomotic leak rate, length of stay (LOS), 90-day readmission rate, and direct medical costs were used for the decision analysis model parameters. A budget impact analysis was conducted to compare the total hospitalization expenditure between ECP and manual circular staplers from the hospital’s perspective in China.

**Results:**

Assuming 100 procedures per year, the anastomotic leak rate was 1.79 and 29.76 per 100 procedures in the ECP and MCS cohorts, respectively. LOS was 1,426.91 days in the ECP cohort, compared to 1,702.38 days in the MCS cohort. The 90-day readmission rate was also lower in the ECP cohort than the MCS cohort (19.10 vs. 26.19 per 100 procedures). For the 100 procedures, the annual total hospitalization costs for left-sided colorectal anastomosis were reduced from ¥7,152,251 using manual circular staplers to ¥6,919,306 using ECP. Despite a higher acquisition cost of ECP compared to the manual staplers (¥711,200 vs. ¥441,700), an annual saving of ¥232,945in the total cost resulted from lower rates of complications and shorter LOS. Sensitivity analyses presented consistent savings using ECP, and the ECP cost and cost of the index hospitalization with anastomotic leak were found the most influencing factors.

**Conclusions:**

There were clinical and economic benefits of ECP, compared to manual circular staplers for left-sided colorectal anastomoses. Further direct comparative studies on the use of ECP in practice in Chinese hospital settings are warranted.

## Introduction

Manual circular staplers have been used as the standard of care for colorectal anastomoses when performing surgical reconstruction, yet its optimal use relies on surgeons’ operation consistency and stability during application [1, 2]. The powered stapling system instead uses battery packs, providing consistent force when firing, and particularly offers help to surgeons with smaller glove sizes [1, 2]. Since the first powered circular stapler, the Ethicon Circular Powered (ECP) Stapler became available in 2019, it has demonstrated less movement during stapler placement and higher leak resistance [3]. The use of ECP in practice was also associated with better clinical outcomes and few technical issues for the creation of left-sided anastomoses [4, 5]. Major postoperative complications of surgical colorectal resection include anastomotic leak, bleeding, ileus, and infection, among which anastomotic leak is the most concerning and occurs in 0.5%-26% of cases [6, 7]. Anastomotic leaks in colorectal surgery increase the total clinical and economic burdens such as a 30-day re-admission, postoperative infection, LOS, and hospitalization costs [8]. Pla-Marti et al. in a retrospective study reported a statistically significant lower rate (1.7%) of anastomotic leak among left-sided stapled colorectal anastomosis procedures using ECP, as compared to 11.8% using manual circular staplers [4]. An indirect comparison between the ECP cohort from a single-arm trial and a matched historical cohort of patients who used manual staplers also presented a lower anastomotic leak rate (1.8% vs. 6.9%), as well as other complication rates and 30-day readmission [9]. The economic benefit of ECP is anticipated because of the avoidance of complications and readmission. A US-based economic analysis showed an annual saving of $44,903 for 100 procedures using ECP relative to manual staplers [10].

Because ECP was launched in China in 2020, there is a lack of evidence on the use of ECP in China to inform decisions with both clinical and economic considerations. Therefore, this study aimed to evaluate clinical and economic outcomes of ECP relative to manual staplers in Chinese patients undergoing left-sided colorectal anastomoses using a combination of decision tree modeling and real-world evidence.

## Methods

### Population/materials and data sources

A decision analysis model was constructed to provide clinical and economic estimates of adult patients who underwent left-sided colorectal anastomoses, using either the powered stapling systems or the conventional manual circular staplers. The occurrence of anastomotic leak and 90-day readmission from an indirect comparison was assessed as the clinical outcomes and the decision model parameters for budget impact analysis. Deidentified information on the use of ECP was derived from the single-arm ECP trial (NCT03326895) [5]. Briefly, the ECP trial enrolled 168 adult patients from the USA and Europe, who underwent left-sided colorectal resections with anastomoses using the 29 mm or 31 mm ECP staplers. The MCS cohort consisted of adult Chinese patients from 20 Chinese tertiary hospitals in 13 cities, who received left-sided colorectal resections with anastomoses using manual circular staplers in January-June 2018. Deidentified information on demographic and clinical characteristics of the patients and billing information of relevant treatments/health care usage was available in the China Health Information System (HIS) database.

### Propensity score matching

Propensity score matching (PSM) at 1:1 ratio was employed to adjust for an imbalance between the two cohorts based on age, gender, comorbidities (i.e., diabetes mellitus and hypertension), and surgical approach (open or non-open surgery) [11]. Non-open approaches included laparoscopic and robotic surgery. The anastomotic leak rate, length of stay (LOS), 90-day readmission rate, and direct medical costs were used for the decision analysis model inputs. Absolute standardized mean differences (SMDs) (< 0.10) were utilized to determine whether the two cohorts were comparable after being matched.

### Model structure

The decision analysis model evaluating the use of ECP instead of manual circular staplers for left-sided anastomosis was built in Microsoft Excel® that included anastomotic leak, as well as 90-day readmission for 100 procedures annually. The 90-day readmission rates and the index hospitalization costs with and without anastomotic leak were estimated based on the PSM-matched data for the MCS cohort. The average listing price of all provinces in China (currency exchange rate: US$ 1 = RMB¥ 6.5) was used to calculate the ECP acquisition cost, whereas the national average listing price of the top 5 brands by market share was used for manual circular staplers [12]. Based on the common clinical practice, the anastomotic leak was identified as either of the following records in the claims: (1) Post-operative drainage tube placement of more than 7 days; 2) procedures including ‘drainage’ and ‘wash’. All costs were adjusted to 2020 RMB based on the Consumer Price Index for medical goods [13, 14]. The adjusted anastomotic leak rates of the two cohorts after PSM were used in the model for a budget impact analysis, which was conducted to compare between ECP and manual circular staplers from a hospital’s perspective in China, following the “Principles of Good Practice for Budget Impact Analysis” by the International Society for Pharmacoeconomics and Outcomes Research (ISPOR) [15, 16]. Relative to manual staplers, the incremental acquisition cost of ECP and potential savings due to decreased treatment cost for anastomotic leaks were reported for the budget impact analysis.

### Sensitivity analyses

Probabilistic sensitivity analyses (PSA) using Monte Carlo simulation with 1000 iterations were performed to account for model uncertainty. The stapler costs (ECP and manual stapler), hospitalization costs per procedure with or without an anastomotic leak, and LOS per procedure with or without anastomotic leak followed gamma distribution; while anastomotic leak rates in the ECP and MCS cohorts respectively, and 90-day readmission rate with or without anastomotic leak followed beta distribution. One-way sensitivity analyses (deterministic) were conducted for key parameters, including anastomotic leak rates, stapler costs, and hospitalization costs with/without an anastomotic leak. 95% confidence intervals (CIs) or ± 25% variations (when CIs unavailable) were used.

All data processing and analyses were performed using Stata/MP 16.0 (StataCorp. 2019. Stata Statistical Software: Release 16. College Station, TX: StataCorp LLC). The required Institutional Review Board (IRB) documentation of the confidentiality of patients’ information was received for this study.

## Results

### Clinical outcomes for model parameters

For the indirect comparison using PSM, 168 patients from the ECP trial were matched at a 1:1 ratio to 168 patients in the China HIS database out of 725 adult patients who received left-sided colorectal anastomoses procedures. Characteristics of the two cohorts before and after matching are presented in Table 1. All SMDs at post-matching were smaller than 0.10, indicating a good balance between the two cohorts. After matching, the mean age of both cohorts was 60 years old, and male patients were more than females in the sample (ECP cohort: 52.98%; MCS cohort: 55.95%). The proportion of diabetes (61.38%) was much higher in the MCS cohort before matching than the ECP cohort (14.88%). In contrast, the proportion of hypertension was slightly lower in the MCS cohort (39.17% vs. 45.24%). Open surgery was used more often in the MCS cohort than the ECP cohort (33.66% vs. 11.90%).

**Table 1:** Cohort characteristics before and after propensity score matching

	Before propensity score matching					After propensity score matching				
	MCS cohort		ECP cohort		SMD	MCS cohort		ECP cohort		SMD
N	725		168			168		168		
Age (years), mean/SD	60.08	11.62	59.91	12.98	0.014	60.35	11.57	59.91	12.98	0.036
Female, n/%	275	37.93%	79	47.02%	0.184	74	44.05%	79	47.02%	0.06
Comorbidity										
Diabetes, n/%	445	61.38%	25	14.88%	1.089	26	15.48%	25	14.88%	0.014
Hypertension, n/%	284	39.17%	76	45.24%	0.123	74	44.05%	76	45.24%	0.024
Surgical approach, n/%										
Open surgery	244	33.66%	20	11.90%	0.536	18	10.71%	20	11.90%	0.029

*SD* standard deviation, *SMD* standardized mean difference

Estimates of all relevant parameters are shown in Table 2. There were lower rates of complications and shorter LOS from the indirect comparison between the ECP trial cohort and the MCS cohort from the China HIS database. The anastomotic leak rate was 1.79 and 29.76 per 100 procedures in the ECP and MCS cohorts, respectively. For the 100 procedures, the LOS was 1,426.91 days in the ECP cohort, compared to 1,702.38 days in the MCS cohort (Table 3).

**Table 2:** Estimates of model parameters

Parameters	Base Value	Standard Error	Lower Bound	Upper Bound	Distribution	Source
Anastomotic leak probability with ECP	0.0179	0.0102	0.0134	0.0223	Beta	ECP Trial
Anastomotic leak probability with manual stapler	0.2976	0.0353	0.2278	0.3675	Beta	China HIS Database
Index hospitalization cost without leak, per case	¥61,760.35	1330.12	¥59,126.12	¥64,394.58	Gamma	China HIS Database
Index hospitalization cost with leak, per case	¥79,720.09	2996.89	¥73,697.61	¥85,742.58	Gamma	China HIS Database
Cost of manual stapler	¥4,417.00	563.403	¥3,312.75	¥5,521.25	Gamma	Listing price in China
Cost of ECP	¥7,112.00	907.160	¥5,334.00	¥8,890.00	Gamma	Listing price in China
Length of stay with leak, per case	23.94 days	1.7560	20.41 days	27.47 days	Gamma	China HIS Database
Length of stay without leak, per case	14.09 days	0.5072	13.09 days	15.10 days	Gamma	China HIS Database
Probability of 90-day readmission with leak, per case	0.4400	0.0702	0.2975	0.5825	Beta	China HIS Database
Probability of 90-day readmission without leak, per case	0.1864	0.0359	0.1151	0.2577	Beta	China HIS Database

**Table 3:** Model outcomes comparing manual circular and ECP staplers

	MCS cohort [A]	ECP cohort [B]	Difference (A-B)
Number of procedures	100	100	N/A
Total direct medical cost of index hospitalization	¥7,152,251	¥6,919,306	¥232,945
Total index hospitalization cost for all procedures with leak	¥2,372,621	¥142,357	¥2,230,264
Total index hospitalization cost for all procedures without leak	¥4,337,930	¥6,065,749	¥-1,727,819
Total cost of staplers	¥441,700	¥711,200	¥− 269,500
Hospital stay for all procedures (days)	1702.38	1426.91	275.48
Hospital stay for all procedures with leak (days)	506.66	25.48	481.18
Hospital stay for all procedures without leak (days)	1195.72	1401.43	− 205.71
Number of 90-day readmissions	26.19	19.1	7.09
Number of 90-day readmissions for all procedures with leak	7.79	0.34	7.45
Number. of 90-day readmissions for all procedures without leak	18.4	18.76	− 0.36

Using average listing prices in different provinces in China, the cost of a manual stapler was estimated to be ¥4,417 and ¥7,112 for ECP (Table 2). The total cost of the index hospitalization was calculated as ¥79,720 with anastomotic leak and ¥61,760 without a leak in the China hospital settings.

### Budget impact analysis

Assuming 100 procedures per year, an annual saving of ¥232,945.10 for 100 left-sided colorectal anastomoses procedures was observed despite the higher acquisition cost of ECP (¥711,200) than the manual staplers (¥441,700) (Table 3). The incremental acquisition cost of ECP compared to the manual staplers was offset by savings in hospitalization costs. The annual total direct medical cost of hospitalization for 100 procedures was ¥7,152,251 using manual circular staplers, consisting of ¥2,372,621 spent on 29.76 cases with anastomotic leak and ¥4,337,930 for 70.24 cases without an anastomotic leak. In contrast, the total cost was ¥6,919,306 using ECP, which was mainly from ¥6,065,749 for 98.21 cases without anastomotic leak and attributed to only ¥142,357 for 1.79 cases with an anastomotic leak. The 90-day readmission rate in the ECP cohort was also lower than the MCS cohort (19.10 compared to 26.19 per 100 procedures).

### Sensitivity analyses

**Fig. 1:**
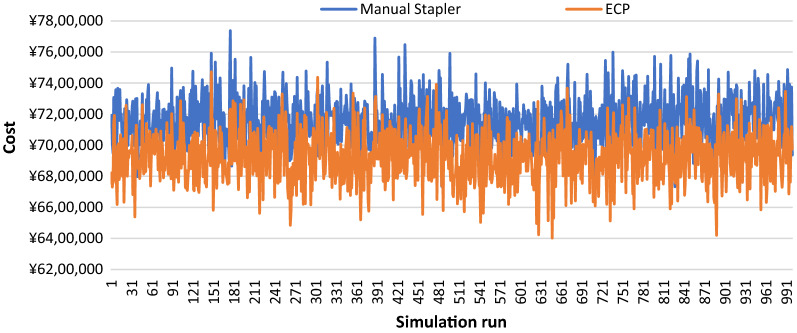
Probabilistic sensitivity analysis: total annual costs for 100 procedures by 1000 simulation runs

**Fig. 2:**
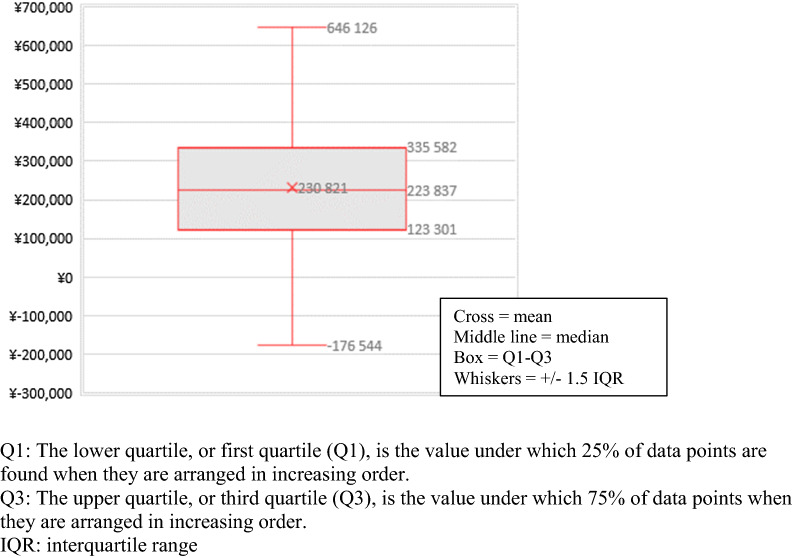
Probabilistic sensitivity analysis: boxplot of annual savings for 100 procedures. Q1: The lower quartile, or first quartile (Q1), is the value under which 25% of data points are found when they are arranged in increasing order. Q3: The upper quartile, or third quartile (Q3), is the value under which 75% of data points when they are arranged in increasing order. *IQR* interquartile range

**Fig. 3:**
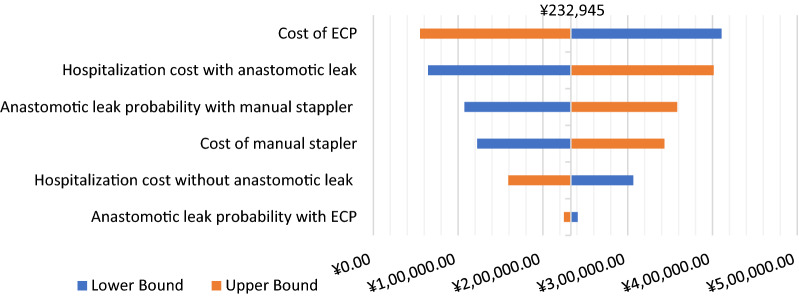
Tornado diagram of annual savings for 100 procedures

Sensitivity analyses presented consistent savings in total cost when comparing ECP with manual circular staplers in the model. The probabilistic sensitivity analysis (PSA) showed that in most cases the costs were larger in the MCS cohort, compared to the ECP cohort (Fig. 1). It also presented the mean saving in the total annual cost of ¥230,821 for ECP relative to manual staplers with the median of ¥223,837 and the interquartile range of ¥123,301- ¥335,582 (Fig. 2). One-way sensitivity analyses showed the total cost savings using the ECP compared to the manual staplers, despite the variations in parameters. ECP cost and hospitalization cost associated with anastomotic leak were identified as the most influencing factors, followed by anastomotic leak rate with manual stapler, cost of a manual stapler, and hospitalization cost with anastomotic leak (Fig. 3). Anastomotic leak rate with ECP was found to have the least influence on cost-saving.

Results were similar using different ratios (1:2 and 1:8) in propensity score matching (results available upon request), yet 1:1 matching yielded the best balance between the groups.

## Discussion

This is the first study on the use of ECP compared to manual circular staplers for left-sided colorectal anastomoses in China. Using several sources of real-world data, this study demonstrated the clinical and economic benefits of ECP over manual circular staplers for left-sided colorectal anastomoses, which were evident in recent studies that however did not involve patients in China [4, 9, 10]. The incremental acquisition cost of ECP replacing manual staplers was ¥269,500, assuming 100 procedures per year; Yet an annual saving in the total cost of the index hospitalization for 100 left-sided colorectal anastomosis procedures using ECP instead of manual circular staplers was estimated to be ¥232,945.10, with 27.98 fewer cases of anastomotic leak and 7.09 cases of 90-day readmissions avoided. This cost-saving was driven by shortened hospital stay by 275.48 days which were also attributed to a lower risk of anastomotic leak. Compared to the previous studies that also utilized the ECP cohort from the ECP trial but US-based data for manual staplers [9, 10], our study showed a larger reduction in cases with an anastomotic leak, length of hospital stay, and the number of readmissions. This mainly resulted from the longer hospital stay and higher complication rates of anastomotic leak in both cases with or without an anastomotic leak in our study. It is commonly known that hospital stay is longer in China than in the US for the same procedures, partially due to fee-for-service payment schemes. The total hospital expenditure in China in the inpatient sector is also driven by the service volume effect [17]. Among the determinants of China’s health expenditure growth, technology was not found to have a significant influencing impact on health expenditure [18]. As another main finding of this study the higher rate of anastomotic leak could be related to a broader definition of anastomotic leak in our study, which is likely to capture more mild cases than those identified by the diagnostic coding system or other clinical symptoms and measures. Due to the lack of specific diagnostic coding for an anastomotic leak in the China HIS database, keyword searching of relevant procedures recorded in billing was used to allow the inclusion of anastomotic leak as much as possible. To be noted, the anastomotic leak rate varies across hospitals/sites and populations [7]. One international census completed in 2010 proposed to identify anastomotic leak as any of the following: (1) Post-operative repeated fever, abdominal pain, and signs of peritonitis; (2) feculent or purulent drainage; (3) Fluid and/or gas collection on CT-scan indicating leak; (4) anastomotic disruption on re-intervention [19]. Using this definition, a single-site retrospective study in China reported an anastomotic leak rate of 4% among 199 patients with rectal cancer undergoing laparoscopic anterior resection from Jan 2016 to April 2019 [20]. Despite the higher anastomotic leak rate for the budget impact analysis, the results of this study appeared valid and robust in China hospital setting according to the sensitivity analyses that found consistent savings of ECP relative to the manual staplers. ECP cost and hospitalization cost with anastomotic leak were found the most influencing, whereas the rate of an anastomotic leak with ECP and manual staplers were the most impacting factors in the US-based study.

Several limitations need to be considered when implicating the results of this study. First, the ECP cohort was from a trial that might not be generalized to other populations/practices [5]. In particular, it was unable to conduct with a parallel control arm, thereby the indirect comparison with a propensity score matching approach was employed in this study. Similar approaches were also used in previous studies [9, 10]. Although statistics indicated a good balance between the ECP and MCS cohorts after matching, unobserved/unknown factors might not be adjusted for. Further studies on the use of ECP in China are warranted once empirical data for a direct comparison study become available. Second, because of the broad definition of anastomotic leak used in this study, the impact of an anastomotic leak might have been aggregated with other complications such as bleeding and infection. Nonetheless, these complications are usually not independent of one another in practice and have been found associated with ECP [5, 9]. Finally, this retrospective data shared some common limitations. The HIS data from the 20 tertiary hospitals in China were analyzed in this study to provide estimates of costs and clinical outcomes for manual staplers that might not represent many other hospitals in China. It needs to be cautious in generalizing our study’s findings to other hospitals in China. Also, the brand and type of manual circular staplers used were not distinguished in the study due to the limited details in the data, yet various brands should have been included as the data were from 20 hospitals in different geographic areas. The indication for surgery was not available in the data, therefore not controlled in the model.

## Conclusions

ECP is favored over manual circular staplers in terms of both the clinical and economic benefits for left-sided colorectal anastomoses in the hospital settings in China. The reductions in complication rates and LOS outweigh the extra acquisition cost of ECP and lead to savings in the total direct medical costs. Future empirical studies for direct comparisons are warranted to assess the use of ECP in China.

## Data Availability

The datasets generated and/or analyzed during the current study are not publicly available but are available from the corresponding author on reasonable request.
